# Identification of Key Genes Involved in Embryo Development and Differential Oil Accumulation in Two Contrasting Maize Genotypes

**DOI:** 10.3390/genes10120993

**Published:** 2019-12-01

**Authors:** Xiangxiang Zhang, Meiyan Hong, Heping Wan, Lixia Luo, Zeen Yu, Ruixing Guo

**Affiliations:** 1 Key Laboratory of Biology and Genetic Improvement of Oil Crops, Ministry of Agriculture, Oil Crops Research Institute, Chinese Academy of Agricultural Sciences, Wuhan 430062, China; zhangxiangxiang@webmail.hzau.edu.cn (X.Z.);; 2 Hubei Province Engineering Research Center for Legume Plants, School of Life Sciences, Jianghan University, Wuhan 430056, China

**Keywords:** embryo, candidate genes, fatty acids, oil accumulation, triacylglycerols

## Abstract

Maize is an important oil seed crop and a major food crop in different parts of the world. Since maize has relatively lower seed oil content as compared to other oil crops, efforts are continuing to improve its oil content percentage. In this study, we analyzed two contrasting maize genotypes with differential oil accumulation percentages. High oil-content (HOC) maize had 11% oil content while low oil-content (LOC) maize had significantly lower oil content (5.4%). Transmission electron microscopy revealed a higher accumulation of oil bodies in the HOC maize embryo as compared to LOC maize. Comparative RNA-sequencing analysis at different developmental stages of the seed embryos identified 739 genes that are constantly differentially expressed (DEGs) at all the six developmental stages from 15 days after pollination (DAP) to 40 DAP. Kyoto Encyclopedia of Genes and Genomes (KEGG) enrichment analysis identified fatty acid metabolism and fatty acid biosynthesis as the most enriched biological pathways contributed by these DEGs. Notably, transcriptional changes are more intense at the early stages of embryo development as compared to later stages. In addition, pathways related to oil biosynthesis and their corresponding genes were more enriched at 30 DAP, which seems to be the key stage for oil accumulation. The study also identified 33 key DEGs involved in fatty acid and triacylglycerols biosynthesis, most of which were up-regulated in HOC, that may shape the differential oil contents in the two contrasting maize. Notably, we discovered that both acyl-CoA-dependent and acyl-CoA-independent processes are essential for the high oil accumulation in maize embryo.

## 1. Introduction

Maize is one of the most important staple food crops and contributes to global food security [1]. It has become the model crop plant to study a wide range of investigations such as crop domestication, genome evolution, epigenetics, heterosis, developmental physiology, quantitative inheritance, and comparative genomics [2]. In addition to its uses as a food and feed source, maize is an important source of vegetable oil because of its relatively large seed size and embryo as well as high quality oil for its higher levels of polyunsaturated fatty acids [3,4,5]. The grain yield of maize is affected by several environmental factors such as drought and temperature stress [6,7,8]. This can also affect the grain oil composition and quality of maize [6,9]. Since it is a quantitative trait, several quantitative trait loci (QTL) were found to control the seed oil accumulation in maize [4,10,11]. Oil in maize seeds mainly exists in the form of triacylglycerol (TAG), so genes related to the TAG synthesis pathway are key regulatory factors in the accumulation process of oil content in corn. Maize seed mainly consists of embryo and endosperm. Embryo inherits the genetic information of the plant and accumulates oil bodies, whereas endosperm is the main food basket for the germinating seedlings [12]. Large embryo size can increase the total oil content in maize seeds but the genetic factors that can enhance oil percentage in embryo may contribute more significantly in the development of high oil content maize. Thus, identification of regulatory mechanisms and biological and molecular processes related to embryo development and fatty acid (FA) and TAG biosynthesis will facilitate the understanding of the mechanism of embryo development and oil accumulation. 

Although maize is an important source of vegetable oil, the oil content percentage of maize grains is relatively low with average of 4%–5% of the seed weight. FA and triacylglycerols (TAG) are the major constituents of seed oil in maize and other grain oils [11,13,14,15]. Oil bodies are intracellular substances composed mainly of TAG molecules which are surrounded by phospholipids [13]. Several genes are proposed to be involved in seed oil accumulation in maize [3]. For example, acyl-CoA:diacylglycerol acyltransferase (*DGAT1-2*), which catalyzes the final step of oil synthesis, significantly increases the seed oil content in maize [16]. Maize *WRINKLED1* encodes a transcription factor belonging to *APETALA2*/ethylene-responsive element binding protein (AP2/EREBP) family that significantly enhances seed oil content in maize embryo via activating the expression of several target genes involved in oil biosynthesis in maize [17]. Several steps of fatty acid biosynthesis are regulated by *WRINKLED1* and expression of the *WRINKLED1* gene has been shown to be under the direct control of the transcription factor *LEAFY COTYLEDON2* (*LEC2*), [18] which is considered, together with *LEC1*, *FUSCA3* (*FUS3*), and *ABA INSENSITIVE3* (*ABI3*), as a master regulator of seed development [19,20,21]. *ZmWRI1* and *ZmLEC1* overexpression have been shown to increase the seed oil content in maize up to 48% [22]. Similarly, overexpression of wheat puroindoline genes (*PINA* and *PINB*) increase germ size and seed oil content in transgenic corn [23]. *ZmSAD1* regulates fatty acid composition and ratio of saturated to unsaturated fatty acids in maize [14]. Several studies on the composition and genetic basis of maize seed oil have been conducted [24,25], but studies regarding molecular identification of key FA and TAG biosynthesis genes and the regulatory network are limited. Thus, identification of genes involved in embryo development, oil accumulation or TAG biosynthesis could significantly improve our understanding of the molecular regulation of oil accumulation and TAG biosynthesis and can contribute to the development of high oil content maize genotypes. 

Transcriptome approaches such as RNA sequencing (RNA-seq) and micro-array have the potential to greatly accelerate the rate of genes’ mining for desired phenotypes [3,26]. Several transcriptome studies on important oil seed crops have identified key candidate genes and molecular pathways related to seed oil biosynthesis [27,28,29,30]. However, transcriptome studies focused on identifying important regulators and processes required for embryo development and/or seed oil biosynthesis in maize based on a genome-wide transcriptional profile are scarce [3,31]. RNA-seq is a more powerful, sensitive and accurate tool for transcriptome analysis than micro-array [32]. RNA-seq analysis identifies the complete set of transcripts which vary according to the phenotype, physiological maturity and tissue type [33]. Thus, transcriptome analysis helps to identify genes which are differentially expressed between two different phenotypes and, thus, aids in understanding the key players of a particular phenotype. 

In this study, we compared the transcriptome profiles between two contrasting maize genotypes (high vs. low oil content) at different developmental stages of seed embryo. Our study identified 739 differentially expressed genes between high oil content and low oil content maize genotypes, which are conserved at all developmental stages of seed embryo. In particular, we identified 33 key DEGs including three novel genes involved in FA and TAG biosynthesis and play role in oil accumulation. Gene Ontology (GO) and Kyoto Encyclopedia of Genes and Genomes (KEGG) analyses revealed key biological processes and molecular pathways related to seed oil metabolism in maize. This work provides insights into the regulation of seed oil biosynthesis in maize and is valuable to the genetic breeding program towards the production of high-quality and -yield maize oil. 

## 2. Materials and Methods 

### 2.1. Plant Materials and Sampling

Two contrasting *Zea mays* inbred lines, 9T159 referred to as high oil-content (HOC) maize and 9T016 referred to as low oil-content (LOC) maize, were obtained from the Oil Crops Research Institute, China. These inbred lines were selected from screening of 300 maize inbred lines for determination of grain oil content, better plant type and more consistent growth. Both genotypes have the same plant type and the same growth period. They were grown in a complete randomized block design with five replications at the research station of the Oil Crops Research Institute, Wuhan, China, in 2018. Developing seeds of these lines were collected in triplicate 30 days after pollination (DAP) for the determination of oil contents following descriptions of Zheng et al. [18]. For transcriptome analysis, developing seeds were collected manually at 15-, 20-, 25-, 30-, 35- and 40-DAP in triplicate. Seeds were soaked in water overnight at room temperature and embryos were then dissected from their endosperm and frozen immediately using liquid nitrogen and stored at −80 °C until use.

### 2.2. Simple and Transmission Electron Microscopy

Embryos at 30 DAP were collected from developing maize seeds and photographed using stereomicroscope. To observe the accumulation of oil bodies in embryo, transmission electron microscopy (TEM) was performed as described earlier [34]. The collected embryos were fixed in 2.5% glutaraldehyde (pH = 7.2) and vacuumed fully to sink at the bottom. Subsequently, samples were successively washed three times with 0.2 mol/L sodium cacodylate buffer for 30 minutes, fixed in 10% osmic acid for 1 h, distilled three times with deionized water for 45 minutes, dehydrated with ethanol, treated with acetone and embedded in epoxy resins and polymerized at 70 °C. The samples were then cut into ultra-thin sections using ultramicrotome and stained by the mixture of uranyl acetate dihydrate and led citrate. The sections were washed with deionized water and visualized using a HITACHI Transmission Electron Microscope (HT7700).

### 2.3. RNA Extraction, cDNA Library Construction, and High-Throughput Sequencing

Thirty-six libraries representing the embryos from the two genotypes collected at six developmental stages and in three replicates were constructed for transcriptome sequencing. Total RNA was extracted using a TIANGEN RNA Prep Pure Plant kit (Tiangen Biotech Co. Ltd, Beijing, China), and purified with the Dynabeads^®^ Oligo (dT)25 kit (Life Technologies, Carlsbad, CA, USA). RNA concentration was measured using nanodrop spectrophotometer (Nanodrop Technologies, Santa Clara, CA, USA) and integrity was evaluated with the Agilent 2100 Bioanalyzer (Agilent Technologies, Santa Clara, CA, USA). The cDNA library was constructed by using a NEBNext^®^ Ultra RNA Library Prep Kit, the quality of retrieved cDNA was checked using the Agilent 2100 Bioanalyzer (Agilent Technologies, USA). After adenylation of 3′ ends of DNA fragments, NEBNext Adaptor with hairpin loop structure were ligated to prepare for hybridization. In order to select cDNA fragments of preferentially 250–300 bp in length, the library fragments were purified with AMPure XP system (Beckman Coulter, Beverly, MA, USA). Then, 3 μL USER Enzyme (NEB, Ipswich, MA, USA) was used with size-selected, adaptor-ligated cDNA at 37 °C for 15 minutes followed by 5 minutes at 95 °C before polymerase chain reaction (PCR). Then PCR was performed with Phusion High-Fidelity DNA polymerase, Universal PCR primers and Index (X) Primer. Finally, PCR products were purified (AMPure XP system) and library quality was assessed on the Agilent Bioanalyzer 2100 system. Sequencing was performed using an Illumina HiSeq™ 2500 paired-end sequencing system.

### 2.4. Sequences Analysis and Assembly

Sequencing-received raw image data were transformed by base calling into sequence data. The raw data was submitted to National Center for Biotechnology Information Sequence Read Archive (SRP217793). Low quality reads and reads with adaptors or N% more than 5% were removed before data analysis using the FastQC tool (http://www.bioinformatics.babraham.ac.uk/projects/fastqc/). After filtering, the remaining high-quality reads “clean reads” stored in FASTQ were used for statistical analyses. The clean reads were then mapped to the *Zea mays* genome (ftp://ftp.ncbi.nlm.nih.gov/genomes/genbank/plant/Zea_mays/latest_assembly_versions/GCA_000005005.6_B73_RefGen_v4) using the TopHat2 tool [35]. The genomic localization results of all sequencing reads data were assembled with the cufflinks package [36], and then were compared to the known genetic models (i) to identify any new genes and alternative splicing events using the cuffcompare package [36], (ii) to discover new exon regions of known genes, and (iii) to optimize the initiation and termination positions of known genes. New genes were aligned by BLASTX (*E*-value < 1 × 10^−5^) to the NCBI nonredundant, Gene Ontology [37], Swiss-Prot [38], KOG [39], and KEGG [40] protein databases to annotate the functions. The gene expression level was determined according to the number of fragments per kilobase of exon per million fragments mapped (FPKM).

### 2.5. Differential Expression of Unigenes

The DESeq package [41] in the R software environment was used to analyze differential gene expression between developing stages and between LOC and HOC. *p*-values were adjusted for multiple testing using the Benjamini–Hochberg false discovery rate (FDR) correction (*p* < 0.05). *p*-value < 0.05 and absolute value of the log2ratio > 0.5 were used as the threshold to determine significant differences in gene expression.

### 2.6. Gene Ontology (GO) and Kyoto Encyclopedia of Genes and Genomes (KEGG) Enrichment Analyses 

GO and KEGG enrichment analyses of the differentially expressed genes were carried out using the Blast2GO software [42]. Enriched p-values were calculated according to the hypergeometric test. All *p*-values were adjusted with the Bonferroni correction. We selected the corrected *p*-value of 0.05 as the threshold to determine significant enrichment of the gene sets. For KEGG Orthology enrichment analysis, we used the false discovery rate 0.05 as the threshold to determine significant enrichment of the gene sets as described before [43].

### 2.7. Quantitative Real-Time Polymerase Chain Reaction (RT-PCR) Analysis

Total RNA was isolated from frozen embryos of different developmental stages. cDNA was synthesized with 1μg total RNA using PrimeScript^®^ RT reagent Kit With gDNA Eraser (TaKaRa, Tokyo, Japan) according to manufacturer’s instructions. Quantitative real-time PCR (qRT-PCR) was performed with SYBR^®^ Premix Ex TaqTM (Perfect Real Time) (TaKaRa, Japan). Gene transcript levels were quantified in triplicate by real-time PCR with the Roche Light Cycler 480 instrument (Roche, Mannheim, Germany). Three biological replicates were analyzed for each sample and the expression level was normalized to that of maize *GAPDH* gene, which was used as the endogenous control gene. The primer sequences used in this study are given in Table S5.

## 3. Results

### 3.1. Characterization of Seed Oil Content and Embryo Phenotype in Contrasting Maize Cultivars

**Figure 1:**
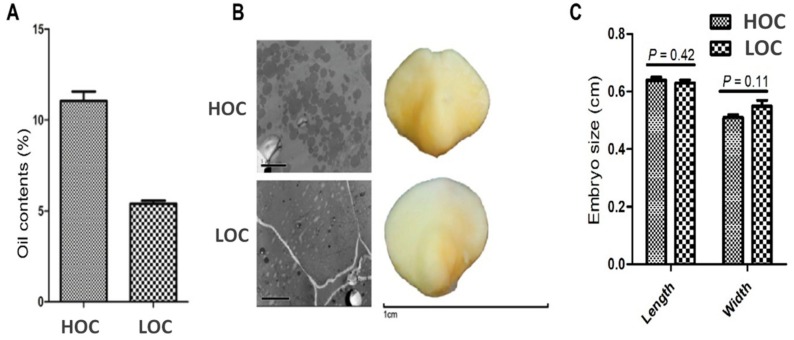
Determination of oil contents in high and low oil contents in maize. (**A**) Quantification of oil contents from seed embryo in high and low oil-content maize. (**B**) Transmission electron micrographs of high and low oil maize embryos. Stereomicroscope images of embryos from high and low oil maize at 30 days after pollination (DAP). (**C**) Quantitative analysis of embryo size (length and width) between high and low oil maize. Values are mean ± standard error (SE) of 5 biological replicates. Significance is shown with *p* value. HOC; high oil-content maize, LOC; low oil-content maize.

In order to elucidate the molecular mechanisms and key genes responsible for oil metabolism and embryo development, we first characterized the two maize genotypes for the oil accumulation and embryo size at 30 days after pollination (DAP). Our results revealed significant difference (*P* < 0.001) in the oil contents of the two maize genotypes (Figure 1a). High oil content (HOC) maize had 11% oil content while low oil content (LOC) maize had 5.4% oil content (almost 50% less) in the seed (Figure 1A). We then observed the accumulation of oil bodies in embryo using transmission electron microscopy. This revealed that more oil bodies were accumulated in HOC maize compared to LOC maize, covering almost the whole cell area (Figure 1B). Since the embryo size has a positive correlation with oil content in maize [10], we tested whether this difference is due to different embryo sizes among the two genotypes. Phenotypic observation of the embryo size using stereomicroscopy revealed that there is no any obvious difference in the embryo size of the two cultivars (Figure 1B). The length and width of the HOC embryo was 0.64 cm and 0.51 cm, whereas, the length and width of the LOC embryo was 0.63 cm and 0.55 cm, respectively (Figure 1C).

Taken together, these results indicate that the high oil content in HOC maize is due to its genetic potential for high accumulation of oil bodies in the embryo, rather than difference in the embryo size.

### 3.2. Generation and Analysis of Dynamic Transcriptome Profiles

**Figure 2:**
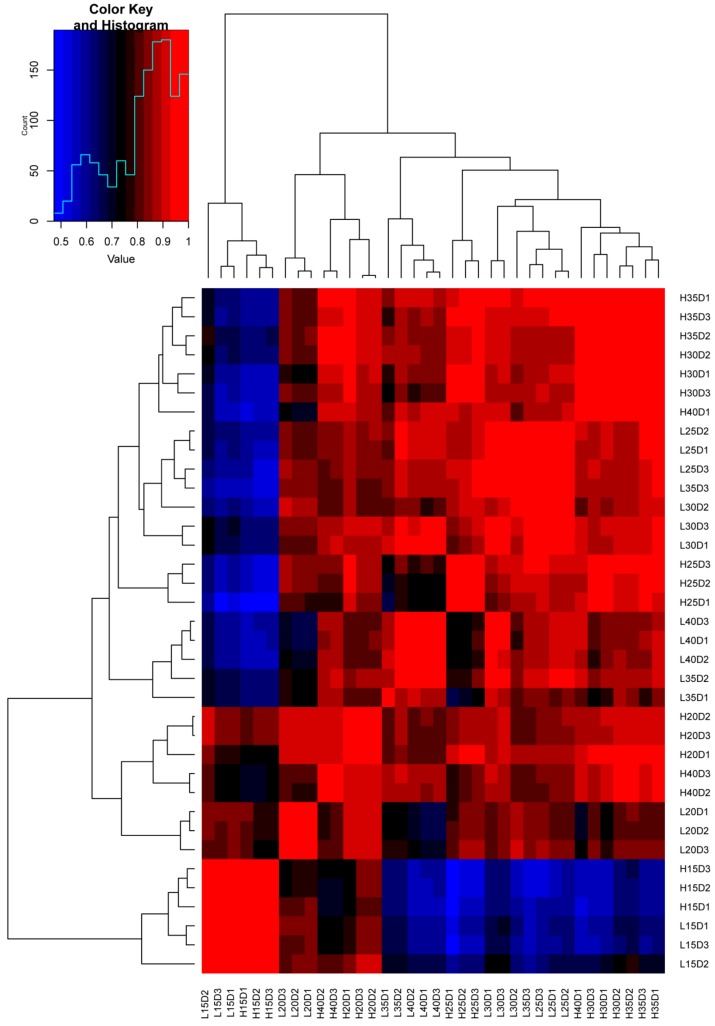
Heat map showing the clustering of differentially expressed genes (DEGs) among different biological replicates at different stages of embryo development. L15D1 represent replicate 1 of low oil maize at 15 days after pollination, and so on. H15D1 represent replicate 1 of high oil maize at 15 days after pollination, and so on. Blue color indicates low expression and red color indicates high expression.

To pinpoint the key genes involved in embryo development and seed oil metabolism, we performed RNA-seq of the embryo samples from the two contrasting maize genotypes (HOC and LOC). RNA was extracted from seed embryo at 15, 20, 25, 30, 35 and 40 DAP and we generated a comprehensive and dynamic transcriptome landscape during embryo development process. Three biological replicates were used for each genotype and at each time point. After sequencing cDNA libraries, total reads were obtained which ranged from 48,995,030 to 82,482,378 (Table S1). Raw reads ranged from 24,541,028 to 41,621,348, while clean reads ranged from 24,497,515 to 41,241,189 (Table S1). Mapping data indicated that more than 85% clean reads were uniquely mapped to the maize reference genome RefGen_v4 (Table S1), thus, indicating a high range of genome coverage of our RNA-seq data. In total, 63,340 unique genes were detected in our RNA-seq dataset. These uniquely mapped reads were used to estimate the gene expression levels using the reads per kilobase per million (RPKM) method. We further detected the events of alternative splicing occurring during embryo development and oil accumulation. The highest number of alternative splicing (AS) events occurred at the transcription start site (TSS) followed by the transcription terminal site (TTS) (Table S2). Furthermore, cluster analysis of the DEGs put different biological replicates together in the heat map, suggesting that biological replicates have high level of similarity (Figure 2). These results indicate that our RNA-seq data was reliable to proceed for further bioinformatics and functional analyses. 

RNA-seq is a useful approach not only to study the global gene-expression profiling of a genome but to identify novel gene transcripts as well [44]. Thus, we attempted to identify novel genes based on transcripts annotation from reference genome RefGen_v4. In total, this study detected 17,005 novel genes annotated on 10 chromosomes (Table S3. In addition, this study has provided the correct structure of 19,793 genes (Table S3). The study has also identified 375,740 novel isoforms of some known genes (Table S3). These results will enrich the genomic information available for maize.

### 3.3. Differential Gene Expression Analysis Related to Embryo Development 

**Figure 3:**
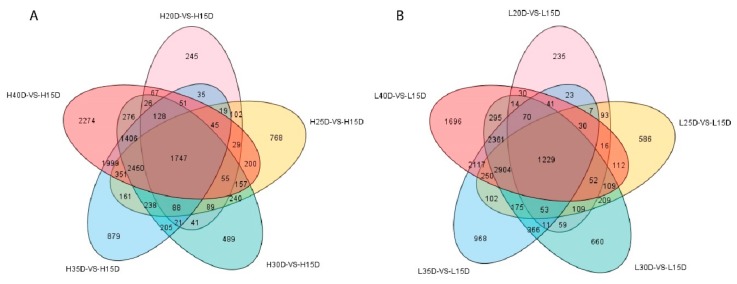
Venn diagram showing genes expressed in each of the five stages of maize in comparison with 15 days after pollination (DAP) in HOC and LOC maize. (**A**) Number of genes expressed between a particular developmental stage compared with 15 DAP and co-expressed genes between different developmental stages in HOC maize. H15D represent developmental stage of 15 days after pollination (DAP) in HOC maize, and so on. (**B**) Number of genes expressed at particular developmental stage in comparison with 15 DAP and co-expressed genes between different developmental stages in LOC maize. L15D represents developmental stages of 15 DAP in LOC maize, and so on. HOC; high oil-content maize, LOC; low oil-content maize.

In order to gain an insight into the genes and biological pathways underlying the embryo development in maize, we identified the constantly and stage-specific DEGs by comparing gene expression from each developmental stage versus 15 DAP (control time point). Concerning HOC maize, we observed 245 DEGs between the stages 15 DAP and 20 DAP, 768 DEGs between 15 DAP and 25 DAP, 489 DEGs between 15 DAP and 30 DAP, 879 DEGs between 15 DAP and 35 DAP, and 2274 DEGs between 15 DAP and 40 DAP (Figure 3A). Notably, most of the DEGs are differentially expressed between the early and last stages of embryo development, suggesting that transcriptional changes are very different among the early and last stages of embryo development (Figure 3A). Furthermore, 1747 DEGs were conserved among all the different stages, which could be key genes for normal embryo development (Figure 3A). Similarly, in the case of LOC maize, 235, 586, 660, 968 and 1696 DEGs were identified at 20, 25, 30, 35 and 40 DAP compared to 15 DAP (Figure 3B). This indicated a similar trend as that of HOC maize. Most of the DEGs were identified at the last stage of embryo development, and 1229 DEGs were conserved among all developmental stages (Figure 3B). Notably, number of DEGs increased over the developmental stages, suggesting that the transcriptional changes are more intense at the later stages of embryo development as compared to early stages, and perhaps the genes governing embryo development are playing major roles at the later developmental phases. We then performed the KEGG analysis to look at the key biological pathways involved in the different developmental stages of seed embryos. Comparative KEGG analysis among different developmental stages indicated that, carbon metabolism, ribosome, fatty acids metabolism, fatty acids degradation and fatty acids biosynthesis pathways were the most enriched biological pathways involved in embryo development in HOC maize (Figure S1). KEGG analysis for LOC maize showed more or less similar results, however, DEGs related to carbon metabolism, fatty acids metabolism, fatty acids degradation and fatty acids biosynthesis are more enriched in HOC maize as compared to LOC maize which may explain its higher oil content in embryos (Figure S2). 

It is worth mentioning that DEGs related to lipid metabolism (fatty acids metabolism, fatty acids degradation and fatty acids biosynthesis) were mostly enriched at 30 DAP, suggesting that 30 DAP is a key stage for oil accumulation (Figure S2). These results will be helpful to understand the key regulatory pathways and pinpoint the key stages of oil accumulation during embryo development in maize seeds.

### 3.4. Differential Gene Expression Analysis Related to Oil Metabolism 

To understand the molecular mechanisms governing oil accumulation patterns in maize, we used GO annotations to classify the enriched DEGs into key biological processes (Figure S3). Overall, single-organism metabolic process, oxidation-reduction process and lipid metabolic process were the most enriched biological processes at all developmental stages (Figure S3). KEGG pathway enrichment analysis indicated that DEGs among HOC and LOC played roles in diverse biological pathways (glycine, serine and threonine metabolism, pyruvate metabolism, peroxisomes, cysteine and methionine metabolism, inositol phosphate metabolism, carbon metabolism glycolysis/gluconeogenesis, cysteine and threonine metabolism, citrate cycle and fatty acid metabolism) in a stage-dependent manner (Figure S4). Since pathways related to fatty acid metabolism, fatty acids degradation and fatty acids biosynthesis are most relevant to oil accumulation, it is worthy to mention that genes in these pathways are mostly enriched at 30 DAP stage specifically. These results further support the premise that 30 DAP is a key stage for oil accumulation and specific DEGs at this stage should be further excavated to understand the molecular mechanism underlying the differential oil accumulation (Figure S4).

**Figure 4:**
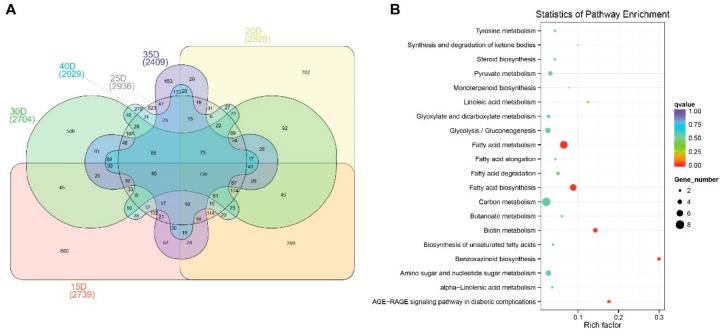
Identification of 739 core genes which are constantly differentially expressed between HOC and LOC at all developmental stages (**A**) and Kyoto Encyclopedia of Genes and Genomes (KEGG) pathway analysis of the core DEGs (**B**).

Furthermore, we compared the DEGs between HOC and LOC at all developmental stages. We obtained 739 core DEGs that are constantly differentially expressed between HOC and LOC maize at all stages (Figure 4A), which may represent the key genes related to the differential oil accumulation among HOC and LOC (Table S4). KEGG pathway enrichment analysis for these 739 core DEGs showed that they mainly contributed to the pathways related to oil metabolism (fatty acid metabolism, fatty acid biosynthesis, biosynthesis of unsaturated fatty acids, carbon metabolism, biotin metabolism), which further indicated that these are the key pathways and DEGs involved in oil accumulation in maize seed embryo (Figure 4B). 

Among these 739 DEGs, 19 DEGs were not expressed in LOC maize at all the embryo developmental stages from 15 DAP to 40 DAP (Table S4). These DEGs could be key genes which are involved in oil accumulation in maize embryo in a positive way (Table S4). Similarly, 21 DEGs were not expressed in HOC maize at all the embryo development stages and, thus, these genes could be negatively regulating oil accumulation in maize (Table S4). 

### 3.5. Differential Expression Analysis of Genes Involved in Fatty Acids (FA) and Triacylglycerols (TAG) Biosynthesis

**Figure 5:**
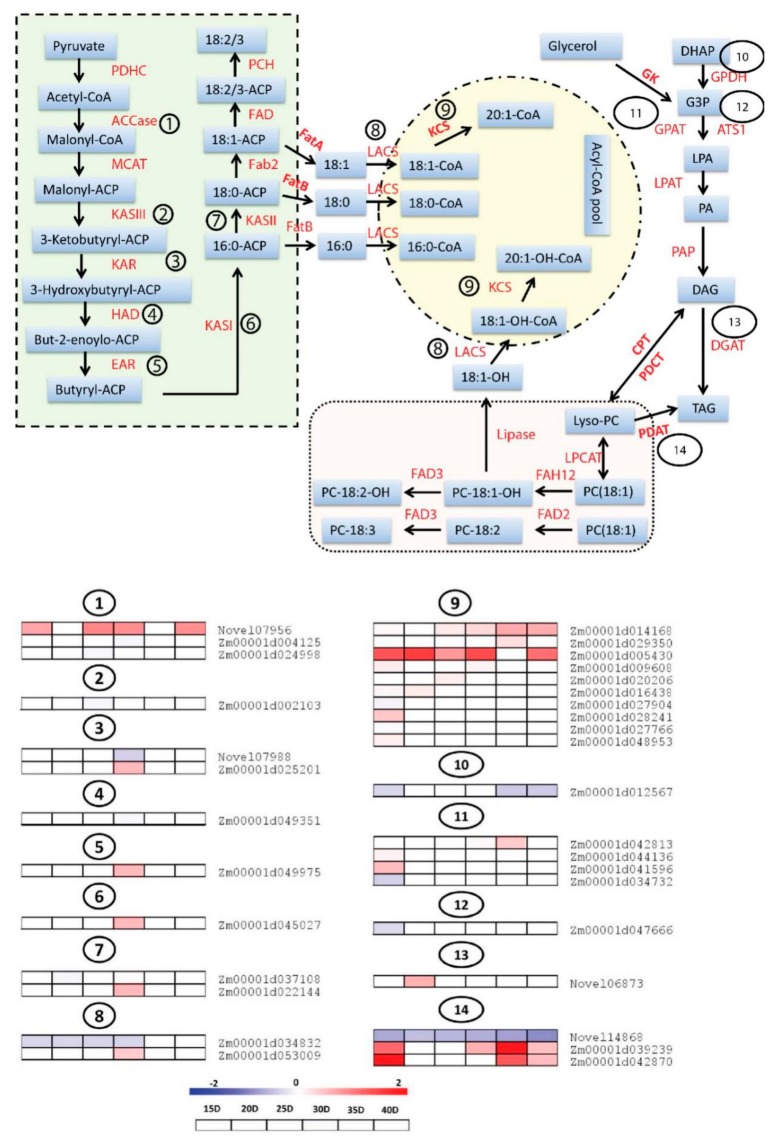
Temporal expression pattern for the genes involved in fatty acid and triacylglycerols (TAG) biosynthesis pathway in maize. The 33 DEGs encoding these enzymes identified from functional unigenes annotation were used to produce the schematic diagram. The DEGs encoding enzymes at each step in the pathway are listed at the bottom of pathway opposite to the number in the circle. The expression pattern of DEGs at 15, 20, 25, 30, 35 and 40 DAP is represented by heatmap. Red (upregulation), blue (downregulation) and white (no significant difference) as indicated in color scale at the bottom of figure. The identified key enzymes involved in lipid metabolism include acetyl-CoA carboxylase carboxyl transferase, (ACCase, EC:6.4.1.2); Malonyl-CoA-ACP transacylase, (MCAT, EC:2.3.1.39); 3-Ketoacyl ACP synthase III, (KAS III, EC: 2.3.1.180); 3-Ketoacyl ACP reductase, (KAR, EC:1.1.1.100); 3R-hydroxymyristoyl ACP dehydrase, (HAD, EC:4.2.1.-); enoyl-ACPreductaseI, (EAR, EC:1.3.1.9); 3-Ketoacyl ACP synthase I, (KASI, EC: 2.3.1.180); 3-Ketoacyl ACP synthase II, (KASII, EC: 2.3.1.180); fatty acyl-ACP thioesterase A, (FatA, EC:3.1.2.14); fatty acyl-ACP thioesterase B, (FatB, EC:3.1.2.14 3.1.2.21); long-chain acyl-CoA synthetases (LACS); 3-ketoacyl-CoA synthase (KCS); glycerol kinase, (GK, EC:2.7.1.30); glycerol-3-phosphate acyltransferase, (ATS1/GPAT, EC:2.3.1.15); lysophosphatidyl acyltransferase, (LPAT, EC:2.3.1.51); phosphatidate phosphatase, (PAP, EC:3.1.3.4); diacylglycerol O-acyltransferase 1, (DGAT1, EC:2.3.1.20); phospholipid: diacylglycerol acyltransferase, (PDAT, EC:2.3.1.158); lysophosphatidylcholine acyltransferase.

Fatty acids (FA) and triacylglycerols (TAG) are the major constituents of seed oil in diverse plant species [26,45]. Different enzymes are involved in the FA and TAG biosynthesis pathway in a series of reactions. Identification of genes encoding these enzymes is crucial to understand the mechanism of oil metabolism in seeds. In this study, we identified 33 DEGs encoding the enzymes involved in FA and TAG pathway and mapped them in order to observe their differential expression at different embryo developmental stages (Figure 5). Acetyl-CoA carboxylase carboxyl transferase, (ACCase, EC: 6.4.1.2) is the first enzyme involved in FA biosynthesis pathway which catalyzes the acetyl-CoA to form malonyl-CoA. Three DEGs (*Novel07956*, *Zm00001d004125* and *Zm00001d024998*) encoding ACCase were identified showing differential expression between HOC and LOC maize. Among these DEGs, *Novel07956* (acetyl-CoA carboxylase) showed strong upregulation in HOC maize at different developmental stages particularly at 15, 25, 30 and 40 DAP (Figure 5). The DEG (*Zm00001d002103*), encoding KASIII was differentially expressed at 25 DAP. We identified two, one, one, one and two DEGs, encoding 3-Ketoacyl ACP reductase (KAR), 3R-hydroxymyristoyl ACP dehydrase (HAD), enoyl-ACPreductase I (EAR), 3-Ketoacyl ACP synthase I (KASI) and 3-Ketoacyl ACP synthase II (KASII), respectively that are differentially expressed at 30 DAP (Figure 5). Among these, only *Novel07988* was downregulated while others were upregulated in HOC maize. Since most of these DEGs showed differential expression at 30 DAP, this suggests that this is a key stage for FA biosynthesis. 

Fatty acyl-ACP thioesterase A, (FATA) and fatty acyl-ACP thioesterase B, (FATB) play key roles for the synthesis of free fatty acids by making the fatty acids free from the acyl carrier protein (ACP). Then, long-chain acyl-CoA synthetases (LACS) adds the CoA group with 16:0, 18:0, 18:1 and 18:1-OH fatty acids to help convert them into an acyl-CoA pool. Two DEGs (*Zm00001d034832* and *Zm00001d053009*) encoding LACS were identified in this study. *Zm00001d034832* showed constitutive downregulation from 15 to 30 DAP while *Zm00001d053009* showed upregulation at 30 DAP (Figure 5). We also identified 10 DEGs encoding 3-ketoacyl-CoA synthase (KCS) which showed differential expression at different embryo development stages. All of these 10 DEGs showed upregulation in HOC maize suggesting that these DEGs positively regulate FA biosynthesis and oil accumulation (Figure 5). 

Glycerol kinase (GK) and Glycerol-3-phosphate dehydrogenase (GPDH) start the initiation of TAG biosynthesis from FA. GPDH catalyzes the reversible redox conversion of dihydroxyacetone phosphate (DHAP) to glycerol 3-phosphate (G3P) [46]. GPDH serves as a major link between carbohydrate metabolism and lipid metabolism. G3PP was subsequently catalyzed to form lysophosphatidic acid (LPA) by glycerol-3-phosphate acyltransferase (GPAT) and ATS1. One DEG each for GPDH and ATS1 was found to be downregulated in HOC maize at different developmental stages of embryo. In addition, four DEGs encoding GPAT were identified which showed differential expression at multiple stages, most of them are up-regulated in HOC maize (Figure 5). Finally, diacylglycerol O-acyltransferase 2 (DGAT) encoded by *Novel06873* converts the diacylglycerols (DAG) into the TAG molecule and *Novel06873* was found strongly up-regulated in HOC maize. 

Regarding acyl-CoA independent pathway for TAG biosynthesis, we identified 3 DEGs encoding PDAT, 2 of which (*Zm00001d039239* and *Zm00001d042870*) were strongly up regulated in HOC maize at different embryo developmental stages, suggesting that both acyl-CoA-dependent and acyl-CoA-independent process are essential for the high oil accumulation (Figure 5). Distinctly, the third DEG encoding PDAT enzyme was a novel gene, *Novel14868*, and was strikingly down regulated throughout all embryo developmental stages in HOC maize, indicating a negative role in high oil accumulation. Taken together, our results showed that up-regulation of many DEGs encoding putative enzymes from the FA and TAG pathway may be the key mechanism of higher oil accumulation in HOC maize. Several detected enzymes have multiple copies with contrasting expression patterns among HOC and LOC, thus complicating a clear understanding of the mechanism leading to the differential oil accumulation. Nonetheless, important DEGs such as *EAR*, *KASI*, *DGAT2* were detected in single copy and could represent potential targets to control the oil accumulation in maize seeds. 

**Figure 6:**
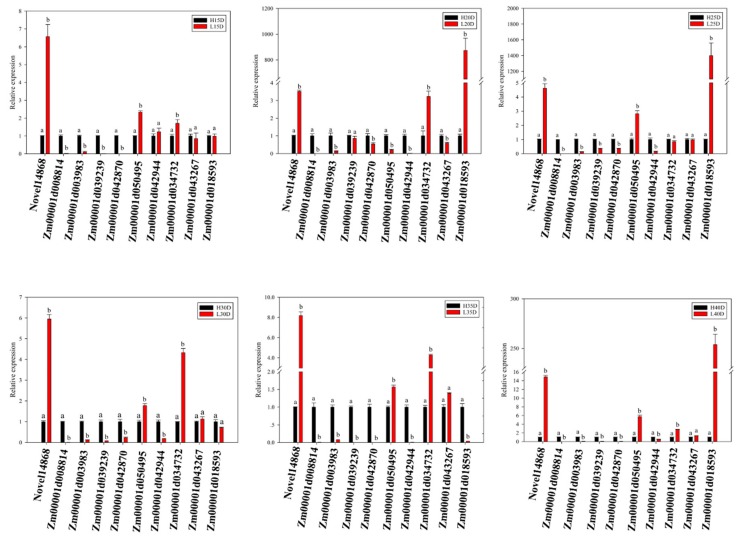
Validation of RNA-seq data by qRT-PCR using 10 differentially expressed genes between high and low oil maize. Values are mean ± SE of three biological replicates. Letters a and b represent statistical significant difference at *p* < 0.05.

In order to validate the reliability of our RNA-seq data, we measured mRNA abundance using qRT-PCR for 10 DEGs from FA and TAG biosynthesis pathways. All the 10 tested genes by qRT-PCR were significantly changed between HOC and LOC maize during the different developmental stages similarly as observed through RNA-seq analysis (Figure 6). These results further consolidate the reliability of our RNA-seq data and the conclusions derived from this data.

## 4. Discussion

Embryo, fatty acids and TAG have the fundamental role in determining the seed oil content and this phenomenon is conserved across different crop species [45]. In this study, we found that the difference in the oil accumulation of the two maize genotypes was due to their different genetic make-up instead of embryo size. Several genes have been identified which control the oil content percentage and quality in maize suggesting that there is great potential for genetic improvement of maize oil content [10,14,16]. Thus, identification of new genes controlling maize oil accumulation would greatly contribute for the development of high oil maize genotypes [5].

Since the contrasting oil phenotypes were due to genetic changes, we used these genotypes to compare their transcriptional profiles using RNA-seq analysis at different developmental stages of seed embryo. We observed that genes governing embryo development are playing major roles at the late developmental phases in maize. This observation was in agreement with some earlier studies which also reported that transcriptional changes related to oil accumulation are intense at late embryo developmental stages in maize and brassicas [3,30]. In the case of *Brassica napus*, it was shown that the seed oil content were highest at later stages after pollination which is consistent with transcriptional changes at corresponding time points [30]. These findings suggest that there is some kind of conserved mechanism of oil accumulation among different species including monocots and dicots. Comparison of the transcriptomes of the two genotypes highlighted DEGs related to carbon metabolism, fatty acids metabolism, fatty acids degradation and fatty acids biosynthesis (Figures S1 and S2). These pathways are directly related to oil biosynthesis and can be the basis of the differential oil accumulation in the two genotypes [45]. Notably, DEGs related to lipid metabolism (fatty acids metabolism, fatty acids degradation and fatty acids biosynthesis) are mostly enriched at 30 DAP, suggesting that 30 DAP is a key stage for oil accumulation in maize (Figure S4). Earlier transcriptome studies related to maize embryo development also revealed that transcriptional changes are intense from 25–30 DAP which further strengthen our results that 30 DAP is an important transition phase for embryo development and oil accumulation [3]. 

“FA and TAG biosynthesis pathway” is the key pathway related to oil biosynthesis in seed embryos [26,45]. Based on functional annotation of DEGs, we identified 33 DEGs encoding enzymes involved in the FA and TAG biosynthesis pathways (Figure 5). The TAG biosynthesis pathway is generally divided into two main steps. The first step involves the biosynthesis of Acyl-CoA pool which is composed of fatty acids of different carbon lengths (C16 and C18). ACCase is a key enzyme that controls the flux of carbon into FAs [47]. This study identified three DEGs encoding ACCase including one novel DEG “*Novel07956*” which had strong upregulation at different stages of embryo development (Figure 5). Notably, *Novel07956* was strongly upregulated at 30 DAP in HOC maize, which is consistent with intense transcriptional changes at this stage, suggesting that this gene has some potential regulatory role in oil accumulation in maize. It has been demonstrated that the FA carbon chain length and saturation are regulated by the activities of *FATA*, *FATB*, *LACS* and *KASII* [27,48]. FATA and FATB play key roles for the synthesis of free fatty acids by making the fatty acids free from the acyl carrier protein. FATA hydrolyzes 18:1-ACP into 18:1 (oleic acid) while FATB hydrolyzes 18:0-ACP into 18:0 (stearic acid) and 16:0-ACP to produce 16:0 (palmitic acid). Then, long-chain acyl-CoA synthetases (LACS) adds the CoA group with 16:0, 18:0, 18:1 and 18:1-OH fatty acids to help convert them into acyl-CoA pool. Here, we identified two DEGs encoding LACS and two for KASII. Interestingly, *Zm00001d053009* encoding LACS and *Zm00001d022144* encoding KASII were expressed only at 30 DAP in HOC maize (Figure 5), suggesting their key role in oil accumulation at 30 DAP. Notably, *Zm00001d034832* encoding LACS was downregulated from 15 DAP to 30 DAP in HOC maize, which indicated that it may be a negative regulator of oil accumulation in maize. Further studies using targeted mutagenesis of this gene could further expand our knowledge about the exact role of *Zm00001d034832* in oil accumulation. Glycerol kinase (GK) and glycerol-3-phosphate dehydrogenase (GPDH) start the initiation of TAG biosynthesis from FA. GPDH catalyzes the reversible redox conversion of dihydroxyacetone phosphate (DHAP) to glycerol 3-phosphate (G3P) [46]. G3P was subsequently catalyzed to form lysophosphatidic acid (LPA) by glycerol-3-phosphate acyltransferase (GPAT) and ATS1. One DEG each for GPDH and ATS1 was found to be downregulated in HOC maize at different developmental stages of the embryos. Acyl-CoA-dependent pathway catalyzed by diacylglycerol acyltransferase (DGAT) is considered the major pathway for TAG assembly in oil seed plants [49]. Here we identified a novel DEG *Novel06873* encoding DGAT2which showed more than 3.5-fold upregulation in HOC maize, suggesting its important role in high oil accumulation. Previously, the role of DGAT1-2 in increased oil accumulation has been reported in oil seed crops and maize [50,51]. Taken together, our findings report several novel genes and some known genes that could be key regulatory genes involved in the FA and TAG biosynthesis pathway and play important roles in oil accumulation in maize. 

In addition to the acyl-CoA based pathway for TAG biosynthesis, oil seed crops have an alternate pathway for TAG biosynthesis, which is independent of acyl-CoA pool [28,52]. This is catalyzed by PDAT enzymes which convert phosphatidylcholine (PC) into TAG via polyunsaturated fatty acids. Here, we found 3 DEGs including one novel gene Novel14868 encoding PDAT, which are involved in acyl-CoA independent pathway for TAG biosynthesis (Figure 5). *Zm00001d039239* and *Zm00001d0428703* showed remarkable upregulation at different developmental stages in HOC maize and, thus, positively regulate TAG biosynthesis via PDAT in a acyl-CoA independent pathway. Notably, *Novel14868* showed downregulation at all the six embryo developmental stages in HOC maize, suggesting that it may negatively regulate oil accumulation (Figure 5). Further characterization of these three genes could give a better understanding of the importance of the acyl-CoA independent pathway in high oil accumulation. One key unanswered question in this study concerns the key regulatory genes (transcription factors) targeting the DEGs involved in the FA and TAG pathway. Recently, Zamora-Briseno et al. [53] employed a large-scale transcriptome data from maize seed to perform a weighted gene-co expression analysis in order to identify key transcription factor (TF) involved in protein disorder during seed development. This approach was very promising and could also be applied to our dataset to find out TFs co-expressed with some DEGs, a result which will further deepen our understanding of oil accumulation mechanisms in maize embryos.

## 5. Conclusions

**Figure 7:**
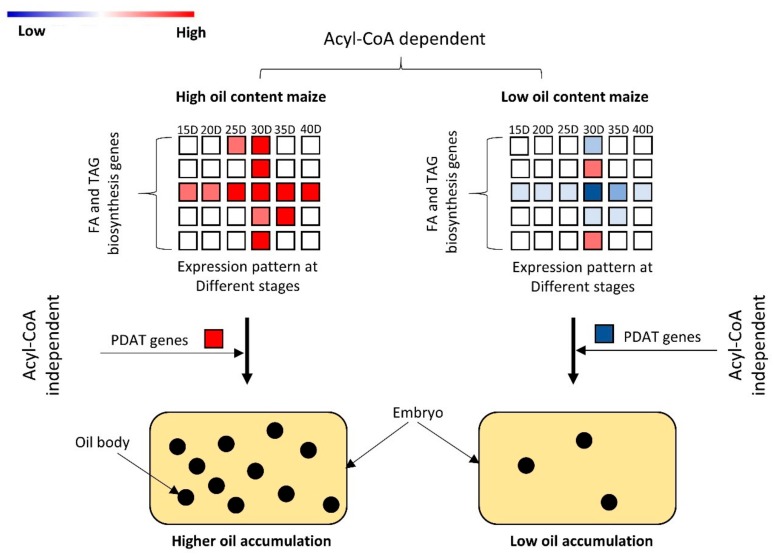
Proposed model for oil accumulation in maize embryo. Two pathways are highlighted in this model. Fatty acyl-CoA dependent pathway is the main pathway for triacylglycerols (TAG) biosynthesis and upregulation of genes encoding enzymes involved in this pathway lead to higher oil accumulation in maize embryos, and vice versa. Fatty acyl-CoA independent pathway is an alternate pathway for TAG biosynthesis. Higher expression of genes encoding PDAT enzyme contributed to higher oil accumulation.

In conclusion, this study has clarified the key stage of oil accumulation in the maize embryo and the underlying molecular pathways (Figure 7). Since FA and TAG biosynthesis is the key pathway for oil accumulation in plants, we have identified 33 DEGs involved in the FA and TAG biosynthesis pathways, which shape the differential oil accumulation in maize embryo. We provide a useful resource for the characterization of these genes for further validation of their function using over-expression and knockout approaches. In addition, this study has generated comprehensive temporal data, which could be harnessed for further analysis in order to identify the key modules and principally the major regulators of the differentially expressed structural genes related to oil accumulation using the gene co-expression approach. 

## Supplementary Materials

Supplementary materials can be found at https://www.mdpi.com/2073-4425/10/12/993/s1. Figure S1. KEGG pathway categories of differentially expressed genes in high oil-content (HOC) maize between 20 days after pollination (DAP) and 15 DAP (A), 25 DAP and 20 DAP (B), 30 DAP and 25 DAP (C), 35 DAP and 30 DAP (D), and 40 DAP and 35 DAP (E); Figure S2. KEGG pathway categories of differentially expressed genes in low oil-content (LOC) maize between 20 days after pollination (DAP) and 15 DAP (A), 25 DAP and 20 DAP (B), 30 DAP and 25 DAP (C), 35 DAP and 30 DAP (D); Figure S3. Gene Ontology (GO) classification of differentially expressed genes (DEGs) at different developmental stages between HOC and LOC at 15 DAP a, 20 DAP b, 25 DAP c, 30 DAP d, 35 DAP e, and 40 DAP f. DEGs are classified into three main categories: biological process, cellular component, and molecular function. H15D represents developmental stages of 15 days after pollination (DAP) in HOC maize, and so on. L15D represents developmental stages of 15 DAP in LOC maize, and so on. * indicates significantly enriched GO terms; Figure S4. KEGG pathway categories of differentially expressed genes between HOC and LOC at 15 DAP a, 20 DAP b, 25 DAP c, 30 DAP d, 35 DAP e, and 40 DAP f. HOC; high oil-content maize, LOC; low oil-content maize. DAP, days after pollination. The ordinate represents the KEGG pathway. The abscissa represents the Rich factor. The larger the Rich factor, the greater the enrichment. The size of bubble indicates the number of differentially expressed genes enriched in the pathway. The redder the color of the bubbles, the more significant the enrichment; Table S1. An overview of sequencing reads from high and low oil maize groups; Table S2. Overview of alternative splicing events in cDNA libraries of high and low oil maize groups; Table S3. Novel transcripts identified in our study; Table S4. Expression analysis of 739 core differentially expressed genes at six different developmental stages of embryo; Table S5. Primer sequences used for qRT-PCR analysis.
